# Effects of a Multicomponent Exercise Program on Physical Functions and Quality of Life in Pre-Frail and Frail Women: A Randomized Controlled Trial Protocol

**DOI:** 10.3390/jcm15218331

**Published:** 2026-10-26

**Authors:** Norah A. Alhwoaimel, Bader A. Alqahtani, Aqeel M. Alenazi

**Affiliations:** Department of Health and Rehabilitation Sciences, College of Applied Medical Sciences in Al-Kharj, Prince Sattam Bin Abdulaziz University, Al-Kharj 11942, Saudi Arabia

**Keywords:** exercise, frailty, physical function, quality of life, randomized controlled trial

## Abstract

**Purpose:** The aim of this study is to assess the effect of a multicomponent exercise program versus usual care on physical performance and quality of life in pre-frail and frail community-dwelling women. **Methods:** A 24-week randomized controlled trial will be conducted on women aged ≥50 years who have pre-frailty or frailty according to the FRAIL scale and are able to walk 10 m without assistance. Recruited participants will be randomized into two groups: the intervention group will receive 24 weeks of multicomponent exercise (12 weeks supervised center-based, then 12 weeks home-based), and the control group will receive their usual care. Physical functions will be measured using Short Physical Performance Battery, Mini Balance Evaluation Systems Test, Activities-Specific Balance Confidence, Falls Efficacy Scale International, Rapid Assessment of Physical Activity, Tilburg frailty instrument, and Barthel Index. Health-related quality of life will be assessed using the Arabic version of SF-12. All outcomes will be measured at baseline and after 12 weeks and 24 weeks of intervention. **Discussion:** Results from this research will show whether 24 weeks of multicomponent exercise will improve physical performance and quality of life among pre-frail and frail women. The findings will guide healthcare providers to effectively monitor and manage frailty in this population.

## 1. Introduction

Frailty is a multidimensional syndrome characterized by a decrease in biological function and increased vulnerability to minor stressors, placing older adults at increased risk for negative health outcomes [1,2]. Fried et al. defined five criteria of a phenotype of frailty as weight loss, exhaustion, low grip strength, slow gait, and low physical activity [1]. The presence of three or more criteria was defined as frailty. Women have higher life expectancy, but also a higher incidence of frailty [3]. In Saudi Arabia, around 18.9% of older adults are frail, and 51.5% are pre-frail [4]. Specifically, women report a higher prevalence of exhaustion (35.3%), weakness (36.2%), and low physical activity (32.8%) [4]. Furthermore, multimorbidity (e.g., hypertension) rises from midlife and peaks in older age, and physical activity declines across late-middle-age and older groups in the Saudi population [5]. These patterns indicate that women ≥50 already accumulate risk and are a priority for early, scalable intervention.

Frailty in the elderly is associated with increased burden of chronic diseases, reduced immune function, and heightened inflammatory responses, which collectively increase their vulnerability to adverse health outcomes such as disability, prolonged hospitalization, and mortality [2,6]. Therefore, early identification and treatment of frailty are crucial in delaying or reversing frailty status among community-dwelling older adults. Research indicates that frailty can be delayed or even reversed with suitable interventions [7,8]. A recent randomized controlled trial among older adults found that combining strength exercises (3 to 5 h per week) with dietary protein intake significantly reduced frailty [9]. Further, a growing body of evidence suggests that exercise-based programs may improve muscle strength, endurance, and balance, addressing key areas impacted by aging and frailty [10,11,12].

In addition, physical activity is a key determinant of healthy aging by reducing biological age through its influence on the determinants of biological aging, which include chronic inflammation and oxidative stress. Recent studies in molecular biology have shown that there is a significant association between higher physical activity levels and lower PhenoAge (b = −0.014, *p* = 0.034) and β2-microglobulin (β2M)levels (b = −0.006, *p* = 0.032), indicating that physical activity promotes healthy aging through molecular and cellular mechanisms [13]. According to the recommendation by the World Health Organisation (WHO), “as part of their weekly physical activity, older adults should do varied multicomponent physical activity that emphasises functional balance and strength training at moderate or greater intensity on 3 or more days a week, to enhance functional capacity and to prevent falls” [14]. In addition, the American College of Sports Medicine (ACSM) recommended that exercise interventions should include a combination of aerobic, strength, balance, and flexibility training [15]. These recommendations lead to improvements in exercise programs related to older adults and specific exercise prescription programs for this population, including VIVIFRAIL©.

VIVIFRAIL© is a low-cost multicomponent exercise program designed by experts in the field of physical exercise and frailty as part of the Strategy for the Promotion of Health and Quality of Life in the European Union to promote physical exercise and prevention of frailty and falls in the elderly [16]. The design of multicomponent exercise was categorized into six different types of programs according to the functional level and risk of falls [17]. Several studies showed the beneficial effect of the VIVIFRAIL program in improving the functional status among older persons with different frailty status [18,19].

A recent systematic review and meta-analysis of 19 randomized controlled trials (RCTs) assessed the impact of multicomponent exercise on physical function of frail community-dwelling elderly [20]. The results showed that 12 weeks of multicomponent exercise significantly improved physical performance, handgrip strength, and knee extensor strength. However, there is insufficient evidence to draw a conclusion about the effectiveness of exercise in enhancing quality of life or long-term health outcomes. Another recent meta-analysis of 10 RCTs showed that multicomponent exercise improves muscle strength, endurance, and balance in frail older adults, and longer interventions (>12 weeks) have a more significant improvement in muscle endurance [11].

Despite the positive effects of multicomponent exercise training, there are only a limited number of studies that have focused on the impact of this type of exercise over a period of 12 weeks or less, with few studies investigating its effects on physical performance in older adults beyond this timeframe [21,22]. Furthermore, the impact of this exercise on quality of life remains unclear [20]. Another challenge in applying multicomponent exercise training is the lack of combining center-based with home-based exercise, which may limit the generalizability and its effect over time beyond the end of programs. The combination of center-based followed by home-based multicomponent exercise programs will facilitate the transition from a supervised program at the center to long-term lifestyle modification. This transition will help in evaluating the feasibility of delivering multicomponent exercise at home and to determine the long-term effect of the exercise program. Therefore, the primary objective of the present study will be to evaluate the effectiveness of a two-phase multicomponent exercise program (12 weeks supervised center-based, then 12 weeks home-based) versus usual care on physical performance and quality of life in pre-frail and frail community-dwelling women. Furthermore, the feasibility of implementing a 24-week multicomponent exercise program will be assessed through adherence and safety. We hypothesize that this integrated approach, involving both home- and center-based multicomponent exercises, will enhance physical performance, improve quality of life, and reduce symptoms of frailty.

## 2. Methods

### 2.1. Study Design

This is a two-arm parallel group, assessor-blinded randomized controlled trial (RCT). The study was designed to determine the effect and feasibility of 12 weeks of center-based multicomponent exercise intervention followed by 12 weeks of home-based exercise in pre-frail and frail women. This study was conducted and reported in accordance with the Consolidated Standards of Reporting Trials (CONSORT) guidelines [23].

The study adhered to the ethical standards of the 1964 Declaration of Helsinki and its subsequent amendments and received approval from the Ethics Committee of Prince Sattam Bin Abdulaziz University (Approval code: SCBR-364/2024) on 15 December 2024. Informed consent was obtained from each participant before the study began. All participant data were anonymized and encoded with a unique identification number to ensure confidentiality. The trial was registered in the Australia New Zealand Clinical Trials Registry (ANZCTR) before the enrolment of participants (Clinical Trial Registration number: ACTRN12625001224415) on the 5 November 2025.

### 2.2. Eligibility Criteria

Inclusion criteria are: (1) community-dwelling women aged ≥50 years; (2) fluent in spoken and written Arabic; (3) have pre-frailty or frailty as defined by the FRAIL scale; (4) able to walk 10 m without assistance; and (5) have not received any rehabilitation during the trial period. The exclusion criteria are: (1) diagnosed with significant neurological conditions, such as Parkinson’s disease or epilepsy; (2) acute lower back or lower limb pain, or severe orthopedic issues that may interfere with the resistance training; (3) severe vision or hearing impairment that prevents normal communication; (4) diagnosed with a major illness (e.g., cancer) or undergone major surgery in the past 6 months; (5) acute stages of various diseases or severe organ failure, such as acute infections, gastrointestinal bleeding, heart failure, respiratory failure, renal failure, etc.; (6) uncontrolled diabetes, hypertension, or heart disease; (7) metal implants such as pacemakers or artificial joints excluded to ensure safety during progressive resistance training; and (8) severe cognitive impairment.

### 2.3. Sample Size

The sample size was calculated to detect a minimal clinically important difference in the Short Physical Performance Battery (SPPB) [24]. Assuming a two-tailed test with an alpha level of 0.05 and a beta level of 0.20 with a power of 80%, a total of 48 participants (24 participants for each group) was needed to detect a difference of at least 1 point on the SPPB, with a pooled standard deviation of 1.19 according to previous research [25]. In a recent systematic review and meta-analysis investigating adherence rates in community-living older adults, the mean adherence was 85% while the retention rate ranged between 72% and 85% for a 24-week program duration [26]. Therefore, considering a 30% dropout rate, the sample size was increased to account for possible dropout. A total sample size of 62 (31 participants for each group) will be recruited. Calculations were performed using (G*Power 3.1.9.4) software.

### 2.4. Recruitment Process and Measurement Procedures

**Figure 1:**
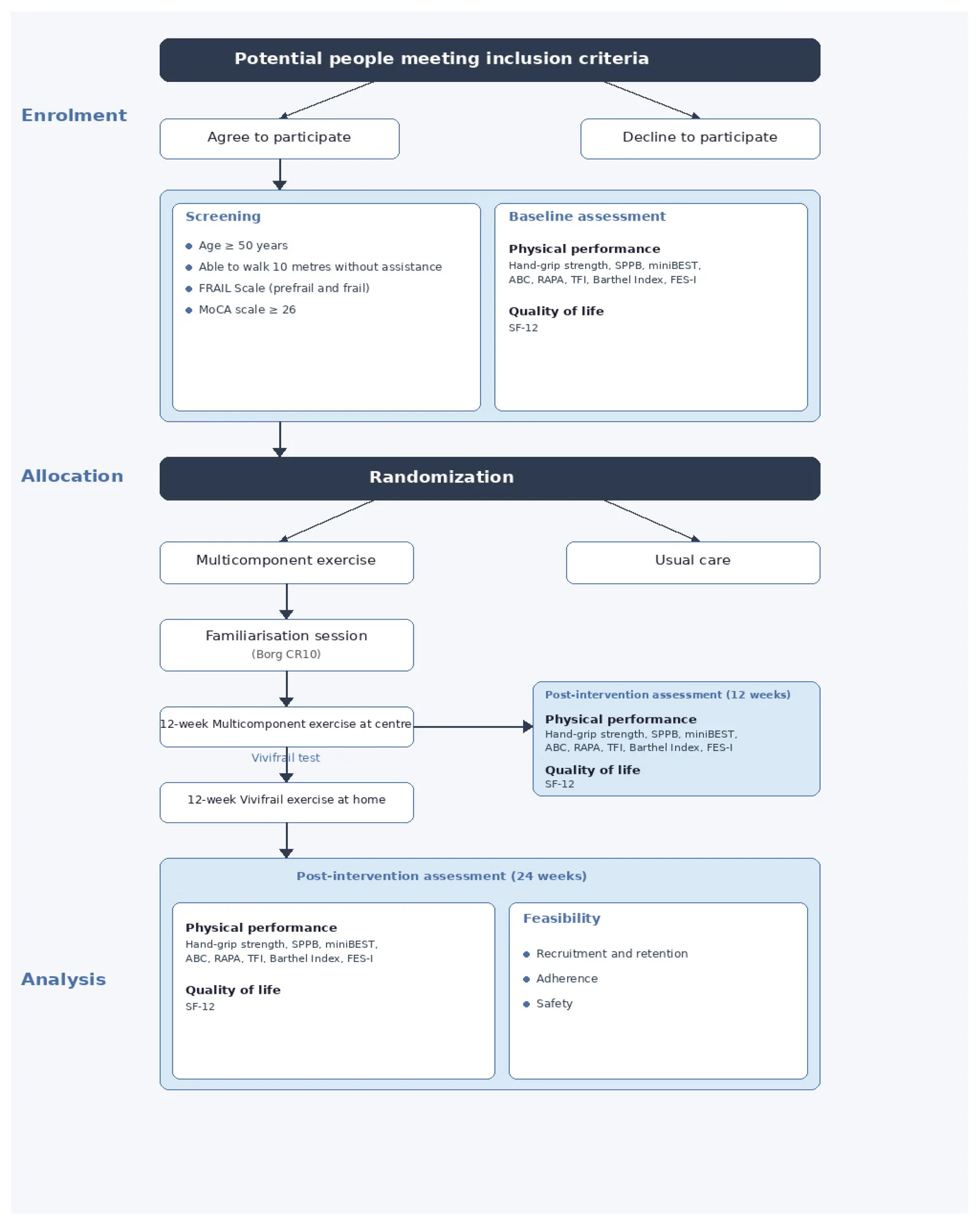
Flowchart of the trial.

An illustrative overview of the outcomes, measures, and timeline is shown in Figure 1. Recruitment was carried out in community service centers in Riyadh city, Saudi Arabia (8 December 2025). Brochures and flyers were distributed to the center visitors; interested volunteers were screened against the eligibility criteria. All the volunteers received a participant information sheet detailing the study information (objectives, measurements, and interventions) and were given an opportunity to ask any questions about the study.

Volunteers were screened by a trained research assistant to assess their eligibility for participation. The screening process involved assessing frailty and cognitive impairment using the Arabic versions of the FRAIL scale (St. Louis University, St. Louis, MO, USA) and the Montreal Cognitive Assessment (MoCA) (MoCA Test Inc., Greenfield Park, QC, Canada) [27,28]. The FRAIL scale included an assessment of five domains: resistance, fatigue, illness, ambulation, and weight loss, with a total score from 0 to 5, with 0 indicating no frailty, 1–2 indicating pre-frailty, and 3–5 indicating frailty [27]. The MoCA scale included eight domains: visuospatial ability, executive function, name recall, memory, attention span, language skills, abstraction, and orientation, with a maximum score of 31. A total MoCA score below 19 indicated severe cognitive impairment [28]. Individuals classified as pre-frail or frail, with a MoCA scale more than 19 and meeting all other inclusion criteria, were eligible to participate in the study.

The current study focuses on women aged 50 years and above, considering previous findings that highlight a high prevalence of frailty-related risk factors such as comorbidities and low physical activity among younger age groups in the Saudi population [4,5].

### 2.5. Randomization and Blinding

After recruitment and baseline measurements, participants were randomly assigned following a 1:1 ratio without restrictions. To ensure allocation concealment, the simple randomization sequence was generated by an independent researcher who was not responsible for recruitment and baseline assessment, using a computer program (www.randomizer.org) to allocate participants to either an intervention group or a control group of usual care. While participants and exercise instructors could not be blinded to the assigned intervention, outcome assessors remained unaware of participant assignment and expected outcomes for each group.

## 3. Interventions

### 3.1. Intervention Group (Multicomponent Exercise)

After randomization, participants in the intervention group took part in a 12-week center-based multicomponent exercise program, followed by 12 weeks of VIVIFRAIL© home-based exercises.

### 3.2. Center-Based Multicomponent Exercise Program

The center-based multicomponent exercise program consisted of 60 min sessions, held three times per week, over a period of 12 weeks. The duration of the exercise program was determined based on the findings of a recent meta-analysis demonstrating that 30 to 60 min of multicomponent exercise, performed 2 to 3 times per week over a 12-week period, significantly improved muscle strength, balance, and endurance in frail older adults [20]. The intervention was delivered by a trained physiotherapist. A detailed exercise component is presented in Table 1.

The resistance exercise included three circuits, and one circuit was performed per session on a rotational basis to ensure balanced full-body conditioning and adequate recovery. The progression of resistance exercises was tailored for each participant using the Rating of Perceived Exertion (RPE) method [29]. The RPE method was considered an optimal method for exercise progression compared to other methods (such as one repetition maximum and repetitions in reserve) as it allowed individuals to complete the exercise set without reaching muscular failure, thereby minimizing the risk of injury and muscle soreness [29].

To determine the starting resistance level (either by weight or TheraBand) for each exercise, participants attended a familiarization session in which they performed three sets of seven repetitions for each exercise. After each set, they rated the difficulty using the Borg CR10 scale [30]. The goal was to begin the exercise program at a moderate level (CR10 = 3–4) for the first two weeks, then progressively increase the intensity to CR10 of 5–7 (strong–very strong). The CR10 scale remained visible to participants throughout the program, and training loads were adjusted when participants consistently reported CR10 of ≤5 on three consecutive days.

Aerobic exercise started at level 3 to 4 on the Borg CR10 scale. The exercise progression of aerobic activities was based on the individual’s tolerance as recommended by ACSM [15]. The Borg CR-10 scale was created as a modification of the original Borg 6–20 RPE scale [30]. It included 11 levels, with values ranging from 0 (representing “no effort at all”) to 10 (indicating “maximum effort during exercise”). The level of exercise was checked every 2 weeks to make sure that participants performed exercise at ≥4 [31]. Participants were instructed to stop exercise at any point if they felt unwell or unable to continue.

### 3.3. Home-Based Multicomponent Exercise Program

After completing the 12-week exercise intervention at the center, participants underwent reassessment and then participated in a 12-week Vivifrail home program. A comprehensive description of the Vivifrail program is available at [http://vivifrail.com/resources/, accessed on 13 September 2024] [32]. The Vivifrail program offers six types of “exercise passports” tailored to the participant’s functional status and fall risk: Passport A for individuals with disabilities, Passport B for those with frailty, Passport B+ for individuals with frailty and fall risk, Passport C for pre-frail individuals, Passport C+ for those with pre-frailty and fall risk, and Passport D for robust individuals. Each passport includes strength, balance, stretching, and walking exercises, and is presented as an “exercise Wheel” [33].

**Figure 2:**
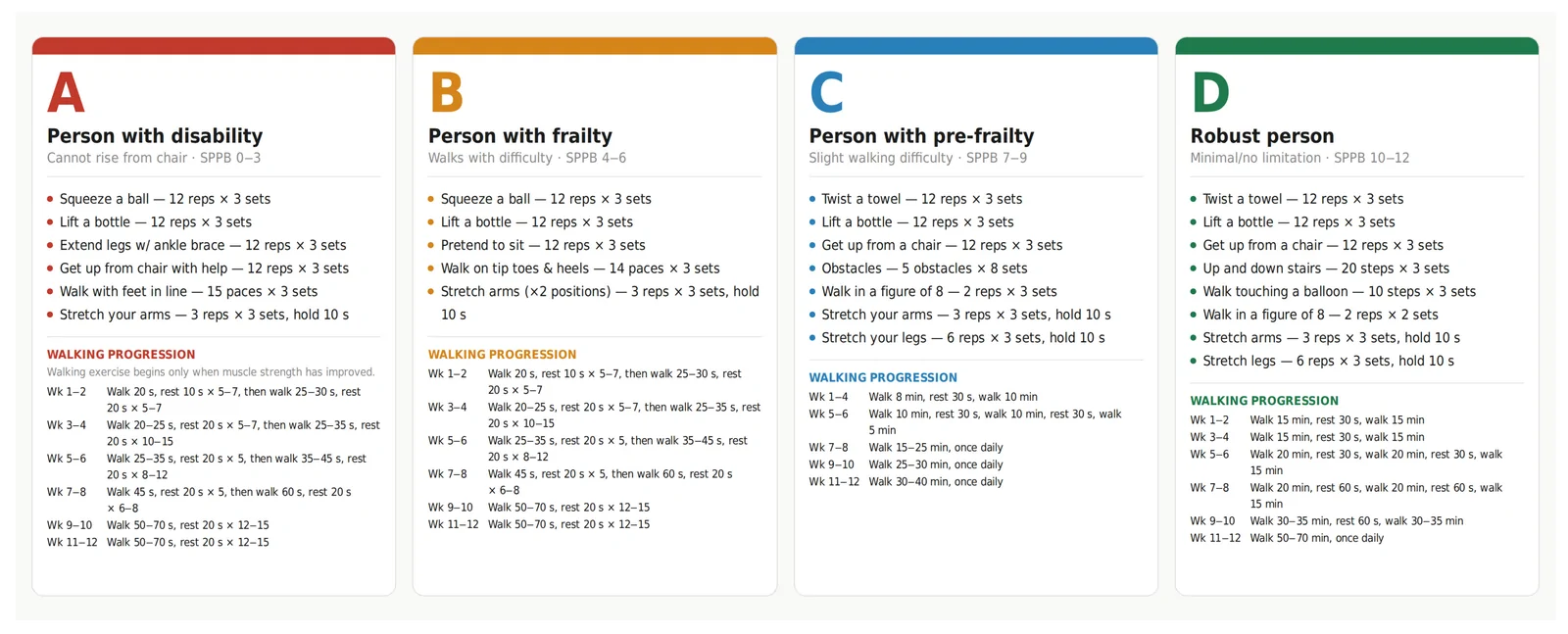
Summary of the VIVIFRAIL multicomponent exercise at home.

Participants were assessed using the Vivifrail test, which included the Short Physical Performance Battery (SPPB) and 6 m walk test to determine their functional level. Based on the results of SPPB, participants were assigned to one of six tailored Vivifrail exercise passports: Disability (of 0–3 points), Frailty (4–6 points), Pre-frailty (7–9 points), or Robust (10–12 points). Each participant received a booklet describing the components of the exercise wheel and how to perform the exercises, and an exercise diary to document their adherence to the Vivifrail home program. Each day that the subject was supposed to exercise, they received reminder text messages from the researcher reminding them to adhere to the planned exercise sessions. The home program of Vivifrail exercise was performed five days per week for 12 weeks, with each session estimated to last approximately 40 to 50 min, with continuous guidance given by a physiotherapist. A summary of the home program for each exercise passport is presented in Figure 2. To make sure that there was a high level of intervention fidelity, all the exercise sessions, either at the center or at home, were supervised by a qualified physiotherapist following a standardized protocol. Adverse events were carefully monitored throughout the study. Minor adverse events such as pain, discomfort, and slight muscle tightness were managed by rest, changes in exercise position, or changes in the degree of exercise intensity. In case of major adverse events such as injuries and serious health problems, the intervention was stopped immediately, and the participant was referred for further medical assessment.

### 3.4. Control Group (Usual Care)

Participants randomly assigned to the usual care group did not receive any specific exercise program and continued their original usual physical activity practiced before the study.

### 3.5. Outcome Measure

Functional capacity (primary outcome) was assessed using the Short Physical Performance Battery (SPPB) (National Institute on Aging, Bethesda, MD, USA), which consisted of three tests: balance, gait speed, and five-chair stand [34]. Balance testing involved standing in three positions (standing with feet side by side, semi-tandem, and full tandem), each held for 10 s. Gait speed was measured by timing a 4 m walk at a usual pace. The five-chair stand measured the time taken to complete standing from a chair five times as quickly as possible. The balance test was scored from 0 to 1 for the side-by-side and semi-tandem positions (10 s = 1 point; <10 s = 0), and from 0 to 2 for the full tandem position (10 s = 2; <10 s = 1; <3 s or unable = 0). The gait speed and five-chair stand subtests were each scored from 0 to 4 (unable = 0; faster = higher). The total SPPB score ranged from 0 to 12, with higher scores indicating better functional performance.

Handgrip strength (HGS) was measured using a handheld dynamometer (GRIPX Digital Hand Dynamometer, China) in a seated position. Participants were instructed to sit with the elbow at a 90° angle, supported by the arm of a chair, and the wrist in a neutral position. Both arms were tested three times, and the average score was used for analysis [35].

Dynamic balance was assessed using the Arabic versions of the Mini Balance Evaluation Systems Test (Mini-BESTest) [36]. The Mini-BESTest consisted of 14 items divided into four domains: (1) anticipatory postural adjustments; (2) reactive postural control; (3) sensory orientation; and (4) dynamic gait. Each item was scored from 0 to 2, with a score of zero indicating an inability to perform the task and a score of two indicating normal performance. The total Mini-BESTest score ranged from 0 to 28, with higher scores indicating better balance.

Balance confidence was measured using the Arabic version of the Activities-specific Balance Confidence (ABC) scale [37]. ABC was a self-reported questionnaire consisting of 16 items to assess confidence in performing daily activities such as “picking up a slipper from the floor” and “walking in a crowded mall”. Responses were rated from 0% (no confidence) to 100% (full confidence), with total scores below 67% indicating balance impairment and increased fall risk [38].

Fear of falling was evaluated using the Arabic version of the Falls Efficacy Scale International (FES-I) (Prevention of Falls Network Europe [ProFaNE], University of Manchester, Manchester, UK) [39]. It was a 16-item self-report questionnaire that included different items on activities of daily living (e.g., walking on slippery surfaces or visiting crowded places), providing information about an individual’s level of concern about falls. Each question had four responses rated from one (not at all concerned) to four (very concerned). The total score ranged from 16 (no concern about falling) to 64 (severe concern about falling).

Physical activity levels were measured using the Arabic version of the Rapid Assessment of Physical Activity (RAPA) (University of Washington Health Promotion Research Center, Seattle, WA, USA). RAPA was a self-reported 9-item questionnaire designed to assess different levels of physical activity, as well as strength and flexibility training [40,41]. The response to each question was either yes or no. The RAPA was divided into two components: RAPA-1, which evaluated aerobic activity across seven items, and RAPA-2, which assessed strength and flexibility training. RAPA assessed the level of physical activity through three levels: light, moderate, and vigorous. The description of each level with graphical examples was included in the instructions. The RAPA1 total score broadly classified subjects into five different levels of physical activity: sedentary, underactive, regular underactive (light activities), regular underactive, and regular active. Responses to RAPA2 were scored one point for strength training, two points for flexibility, or three points for both strength and flexibility.

Frailty was measured using the Arabic version of Tilburg Frailty Instrument (TFI) (Tilburg University, Tilburg, The Netherlands) in conjunction with the FRAIL scale used for initial screening [42,43]. The advantage of the TFI was to assess both physical and cognitive frailty, offering a multidimensional view of frailty that the FRAIL scale alone does not capture. The TFI was a self-administered 15-item questionnaire that covered three domains of frailty, including physical, psychological, and social. The total score ranged from 0 to 15, where higher scores indicated greater frailty, and a cutoff score of ≥5 was used to diagnose frailty.

Activity of daily living (ADL) was assessed using the Barthel Index (BI). BI included 10 items related to basic ADLs, such as bowel and bladder control, grooming, feeding, toilet use, transferring, mobility, dressing, stair climbing, and bathing. Each item was scored between 0 and 15. The total score ranged from 0 (indicating maximum disability and dependency) to 100 (indicating maximum strength and independence) [44,45].

Health-related quality of life (QoL) was assessed using the Arabic version of the SF-12 [46,47,48]. The SF-12 was a self-reported multipurpose measure of QoL derived from the SF-36 and contained only 12 items. The 12 items were divided into two domains: Physical Component Summary (PCS) and the Mental Component Summary (MCS). The PCS focused on measuring the following QoL concepts: general health, mobility, physical activity limitations, stair climbing ability, and work limitations due to physical issues or pain. The MCS focused on emotional aspects such as depression, anxiety, social activity, and the impact of feelings on productivity. The total QoL scores for PCS and MCS ranged from 0 to 100, where higher scores indicated better quality of life.

The feasibility of the study was evaluated through predefined criteria for recruitment, retention, adherence, and safety (adverse events). Recruitment was defined as the proportion of individuals invited to participate who agreed to enroll in the study, while retention was calculated as the percentage of participants who completed the multicomponent exercise program [49]. Recruitment was considered successful when a target sample of participants had been randomized within the enrollment timeframe. Retention was deemed successful if at least 70% of the randomized subjects completed the 24-week multiple exercise intervention, considering the predetermined 30% drop-out rate. Adherence to the exercise program was calculated as the number of sessions attended divided by the total number of planned sessions [50]. The higher levels of adherence were considered as a higher feasibility and acceptance of the program. Safety was monitored during each session by recording any adverse events and asking participants at the beginning of each session whether they had experienced falls, pain, or other symptoms related to the intervention since the last session. Lower adverse events were considered indicative of better feasibility and program safety.

### 3.6. Statistical Analysis

The data were analyzed using the IBM SPSS Statistics, Version 30 (IBM Corp., Armonk, NY, USA). The normal distribution of the data was assessed using the Kolmogorov–Smirnov test. Continuous variables were presented as mean (SD) when normally distributed and as median with interquartile range (IQR) if not. Categorical variables were reported as frequency counts and percentages. Baseline statistical comparisons were conducted using appropriate tests based on the type and distribution of the data: *t*-test or Mann–Whitney U-test for continuous variables, and Chi-squared test for categorical variables. Baseline characteristics were compared between the multicomponent exercise group and the control group to identify the adequacy of randomization and possible confounders. To evaluate the intervention effects across the assessment time points (baseline, 12 weeks, and 24 weeks), linear mixed-effects models were used to analyze the primary outcome measure. Estimated mean differences were reported with 95% confidence intervals and *p*-values. The models accounted for potential baseline differences for the primary outcome measure, adjusting for covariates such as age.

The missing data were handled according to the reasons for missingness. In the case that it was possible to assume that the missing data were missing at random (MAR), the Intention to Treat (ITT) analysis was conducted using multiple imputation, thus including all randomized subjects in the primary ITT analysis. In the case that it was not possible to assume MAR, we considered other more advanced approaches for the primary analysis, taking into account data missing not at random (MNAR).

## 4. Discussion

This paper outlines the protocol for an assessor-blinded RCT to determine the effect and feasibility of combined center- and home-based multicomponent exercise in pre-frail and frail women in Saudi Arabia. This study adds to the literature on the benefits of combining center-based and home-based exercise programs for frail and prefrail women. The findings highlight the long-term feasibility of such a program on comprehensive outcomes including physical functions and quality of life among prefrail and frail women in Saudi Arabia. Recent meta-analysis studies demonstrate that multicomponent exercise contributes to improving muscle strength, balance, and endurance in frail elderly individuals [11,12,20]. However, there is no conclusive evidence of its effect on enhancing quality of life or long-term health outcomes [20]. Just a few trials have investigated the effect of multicomponent exercise on quality of life and ADL among frail elderly [51]. Moreover, the multicomponent exercise was mainly implemented 2–3 times per week and lasted no longer than 12 weeks [20]. Encouraging older adults to recognize the long-term benefits and maintain these exercise habits remains a challenge. Therefore, the results of the current study inform the literature and clinical practice about the long-term effects of multicomponent exercise. Additionally, combining the center program with a home exercise program can be a strategy to promote a healthy lifestyle and sustain exercise habits in older adults.

Several studies have shown that multicomponent interventions can either reverse frailty or slow its progression [52,53]. A recent meta-analysis of 28 RCTs showed a nearly 34% reduction in standardized frailty prevalence, with over 73% fewer individuals classified as frail after completing a 12-week multicomponent exercise program [54]. They recommend that future research should promote the use of frailty measures to build bridges between different definitions of frailty and classify participants into pre-frail and frail populations at study baseline to better understand treatment effects [54]. Thus, the results of this trial include pre-frail and frail participants and consider the classification of participants to better understand the role of exercise in slowing or reversing frailty.

## Figures and Tables

**Table 1:** Multicomponent exercise description.

Exercise Training	Description	Duration	Intensity Progression
**Warm-up**	• Brisk walking • Neck, upper and lower limb stretches	10 min	—
**Strength resistance training**	Each circuit performed in one session. **Circuit 1:** • Shoulder flexion/extension • Hip flexion/hip extension • Ankle plantar flexion/dorsiflexion • Wall push-up • Chair stands **Circuit 2:** • Shoulder abduction/adduction • Biceps curl • Backward arm press • Hip abduction/adduction • Knee flexion/extension • Ankle plantar flexion/dorsiflexion **Circuit 3:** • Shoulder abduction/adduction • Biceps curl • Hip abduction/adduction • Wall push-up • Chair stands	20 min	• 3 sets, 7–10 repetitions • Start at 3–4 on CR10 scale, then progress to 5–7 • Individual training loads adjusted when participant consistently reports CR10 ≤ 5 on three consecutive sessions
**Balance**	**Circuit 1:** • Sideways walking • Heel-toe standing **Circuit 2:** • Tandem walking • Backward walking **Circuit 3:** • One-leg stand • Standing on tiptoes and hold	10 min	Progression according to participant ability: • With support • Without support
**Aerobic**	• Seated marching • Leg marching (standing) • Knee-ups opposite hand touch (sitting) • Step-outs • Step back and forth (sitting) • Stepping jacks	10 min	• Start at 3–4 on Borg CR-10 scale • Progressed every 2 weeks according to participant tolerance
**Cool down**	• Neck, upper and lower limb stretches	10 min	—

## Data Availability

The raw data supporting the conclusions of this article will be made available by the authors on request.
